# Andrographolide, isolated from *Andrographis paniculata* *, *induces apoptosis in monocytic leukemia and multiple myeloma cells via augmentation of reactive oxygen species production

**DOI:** 10.12688/f1000research.53595.1

**Published:** 2021-07-06

**Authors:** Hiroki Doi, Taei Matsui, Johannes M. Dijkstra, Atsushi Ogasawara, Yuki Higashimoto, Seiji Imamura, Tamae Ohye, Hiromu Takematsu, Itsuro Katsuda, Hidehiko Akiyama

**Affiliations:** 1 Field of Clinical Laboratory Sciences, Fujita Health University Graduate School of Health Sciences, Toyoake, 470-1192, Japan; 2 Faculty of Medical Technology, Fujita Health University School of Medical Sciences, Toyoake, 470-1192, Japan; 3 Institute for Comprehensive Medical Science, Fujita Health University, Toyoake, 470-1192, Japan; 4 Department of Hematology, Fujita Health University School of Medicine, Toyoake, 470-1192, Japan

**Keywords:** Andrographis paniculata, andrographolide, apoptosis, reactive oxygen species, monocytic leukemia cells, multiple myeloma cells

## Abstract

**Background**: Andrographolide (Andro) is a diterpenoid component of the plant *Andrographis paniculata* that is known for its anti-tumor activity against a variety of cancer cells.

**Methods**: We studied the effects of Andro on the viability of the human leukemia monocytic cell line THP-1 and the human multiple myeloma cell line H929. Andro was compared with cytosine arabinoside (Ara-C) and vincristine (VCR), which are well-established therapeutics against hematopoietic tumors.

**Results**: Andro reduced the viability of THP-1 and H929 in a dose-dependent manner. H929 viability was highly susceptible to Andro, although only slightly susceptible to Ara-C. The agents Andro, Ara-C, and VCR each induced apoptosis, as shown by cellular shrinkage, DNA fragmentation, and increases in annexin V-binding, caspase-3/7 activity, reactive oxygen species (ROS) production, and mitochondrial membrane depolarization. The apoptotic activities of Andro were largely suppressed by N-acetyl-L-cysteine (NAC), an inhibitor of ROS production, whereas NAC hardly affected the apoptotic activities of Ara-C and VCR. Furthermore, whereas Ara-C and VCR increased the percentages of cells in the G0/G1 and G2/M phases, respectively, Andro showed little or no detectable effect on cell cycle progression.

**Conclusions**: Andro induces ROS-dependent apoptosis in monocytic leukemia THP-1 and multiple myeloma H929 cells, underlining its potential as a therapeutic agent for treating hematopoietic tumors. Notably, the high sensitivity of H929 cells is encouraging for further studies on the use of Andro against multiple myeloma.

## Introduction

Many plant-derived products possess a potential for use in chemotherapy. For example, vincristine (VCR) and vinblastine—two natural alkaloids isolated from *Vinca rosea*—inhibit cell division and are commonly used in anticancer medicine ( Varma *et al.*, 2011). Another example is andrographolide (Andro), a diterpenoid lactone isolated from the Asian herbal plant *Andrographis paniculata*, which has a variety of pharmacological effects including anti-tumor, anti-inflammatory, anti-viral, and anti-malarial activities ( Hao *et al.*, 2020; Kishore *et al.*, 2017; Kumar *et al.*, 2020; Sareer *et al.*, 2014).

Andro has been shown to have anti-tumor activities against solid and hematopoietic tumor cell lines, established from colon-, gastric-, liver-, breast-, and prostatic cancers, leukemia, and lymphoma ( Banerjee *et al.*, 2016; Chen *et al.*, 2012; Cheung *et al.*, 2005; Dai *et al.*, 2017; Khan *et al.*, 2018; Kim *et al.*, 2005; Yang *et al.*, 2010). Common observations in these studies were that Andro reduced the cell viability/proliferation, although the mechanisms were found to differ per cell type. In most cases, the reduced viability of the tumor cell lines could at least partially be explained by the induction of apoptosis ( Banerjee *et al.*, 2016; Cheung *et al.*, 2005; Dai *et al.*, 2017; Khan *et al.*, 2018; Kim *et al.*, 2005; Yang *et al.*, 2010), but in several liver cancer cell lines the cell death caused by Andro was distinct from apoptosis ( Chen *et al.*, 2012). Moreover, among different tumor cell lines, Andro treatment showed a variable effect on the cell distribution among cell cycle phases ( Banerjee *et al.*, 2016; Cheung *et al.*, 2005; Dai *et al.*, 2017; Khan *et al.*, 2018). From the perspective of its potential usage as an anti-cancer drug, the most important observations were that at concentrations at which Andro significantly reduced the viability of tumor cells, normal epithelial cells and lymphocytes were not noticeably affected ( Banerjee *et al.*, 2016; Khan *et al.*, 2018; Yang *et al.*, 2010). Equally important from a therapy point of view was that, at least *in vitro*, primary lymphoma cells were even more sensitive to Andro than lymphoma cell lines ( Yang *et al.*, 2010).

THP-1 (RRID:CVCL_0006) is a permanent human monocytic cell line derived from an acute monocytic leukemia patient ( Abrink *et al.*, 1994; Tsuchiya *et al.*, 1980). Previously, preparations or modifications of Andro were found to be toxic for THP-1 cells ( Habtemariam, 2003; Lee *et al.*, 2012), to enhance the cells’ expressions of cytokine IFNγ and of stress-protein GRP-78 ( Gupta *et al.*, 2020), and to interfere with their functional properties such as the (immune-induced) activation and/or production of transcription factor NF-κB, matrix metalloproteinase-9, and various cytokines ( Gupta *et al.*, 2020; Lee *et al.*, 2012; Low *et al.*, 2015; Nie *et al.*, 2017), and their migration in a chemotaxis assay ( Zhang *et al.*, 2019). An analogue of Andro,14-Deoxy-11,12-didehydroandrographolide (AND2), induced apoptosis in THP-1 cells ( Raghavan *et al.*, 2014), but—to the best of our knowledge—the present study is first to address how Andro itself induces apoptosis in these cells.

H929 (aka “NCI-H929”) (RRID:CVCL_1600) is a permanent human IgA-kappa-producing multiple myeloma cell line ( Gazdar *et al.*, 1986) for which the sensitivity to Andro—as far as we know—has not been investigated. However, Andro was found to have inhibitory/cytotoxic/apoptotic effects on other multiple myeloma cell lines, even at low concentrations ( Gao and Wang, 2016; Gunn *et al.*, 2011). These high sensitivities led us to be interested in the effects of Andro treatment on H929 cells.

In the present study, we investigated the viability-reducing effects and their mechanisms of Andro on both THP-1 and H929 cells. The effects of Andro were compared with those of the common anti-cancer drugs VCR and Ara-C (aka cytarabine). VCR and Ara-C are widely used as chemotherapeutic agents against soft tissue tumors and hematopoietic tumors including acute leukemia, lymphoma, and multiple myeloma ( Koharazawa *et al.*, 2008; Lu *et al.*, 2003; Short and Ravandi, 2016; Tsimberidou *et al.*, 2014). Andro showed an excellent viability-reducing activity against both THP-1 and H929 cells, and in the case of H929 cells this effect was markedly superior to that of VCR or Ara-C. Unlike with Ara-C and VCR, the viability-reducing effect of Andro was found to be dependent on the enhanced production of reactive oxygen species (ROS).

## Methods

### Materials

Andro was purchased from Tokyo Chemical Industry (Tokyo, Japan), dissolved in ethanol at 10 mM, and used at 10-50 μM. Cytosin arabinoside (Ara-C) and vincristine (VCR) were purchased from SIGMA-ALDRICH (Missouri, USA), dissolved in phosphate-buffered saline (PBS; 150 mM NaCl, 10 mM phosphate-buffer, pH 7.2), and used at 40 μM and 0.1 μM, respectively. Carbobenzoxy-valyl-alanyl-aspartyl-[O-methyl]-fluoromethylketone (Z-VAD-FMK), a pan-caspase inhibitor was purchased from Promega (Tokyo, Japan) and used at 20 μM. As an antioxidant, N-acetyl-L-cysteine (NAC) was purchased from Funakoshi (Tokyo, Japan), dissolved in ultra-pure water at 1 M and used at 3 mM.

### Cell culture

THP-1 cells (human monocytic leukemia cell line; EC88081201; RRID:CVCL_0006) and NCI-H929 cells (human IgA-kappa-producing multiple myeloma cell line; EC95050415; RRID:CVCL_1600) were obtained from DS PHARMA BIOMEDICAL (Osaka, Japan). They were grown in RPMI 1640 medium (Sigma-Aldrich) supplemented with 10% fetal bovine serum (FBS; Equitech-Bio Inc, Kerrville, USA), 100 U/mL of penicillin, and 100 μg/mL of streptomycin (GIBCO, Carlsbad, USA) at 37 °C with 5% CO _2_. For experiments, Andro, Ara-C, or VCR were added to the cell cultures at the appropriate concentrations. NAC was added to the cell culture one hour before the addition of Andro, Ara-C, or VCR. Control (untreated) cells were harvested at 24 h.

### Morphological observation

Cells were deposited on glass slides by the cytospin method at 40×g for 5 min (Cyto-Tek 2500 Cytocentrifuge, Sakura, Tokyo, Japan) ( Tokunaga *et al.*, 2017). The glass slides were fixed with Wright’s solution (Muto Pure Chemicals, Tokyo, Japan) and stained with Giemsa’s solution (Muto Pure Chemicals) to observe the morphological changes of the cells.

### DNA fragmentation analysis

Cells were centrifuged at 300×g for 5 min and washed once with PBS. The cell pellet was suspended in 100 μL of cell lysis buffer (10 mM Tris–HCl buffer, pH 7.4 containing 10 mM EDTA and 0.5% Triton X-100), and kept at 4°C for 10 min. Cell lysate was centrifuged at 16,000×g for 20 min. The supernatants (100 μL) were incubated with 2 μL of RNase A (20 mg/mL; MACHEREY-NAGEL, USA) at 37°C for 60 min, and then with 2 μL of proteinase K solution (20 mg/mL; Wako, Japan) at 37°C for 60 min. After adding 20 μL of 5 M NaCl and 120 μL of isopropyl alcohol, these mixtures were kept at −30°C overnight. The precipitate was then collected by centrifugation at 16,000×g for 15 min and washed twice with 70% ethanol. After removal of ethanol, samples were allowed to stand for 5 min on a clean bench to volatilize the remaining ethanol. DNA samples were then dissolved in TE buffer (10 mM Tris–HCl, pH 7.4 and 1 mM EDTA), and subjected to 2% agarose gel electrophoresis at 100 V for 45 min. DNA was stained with 0.5 μg/mL ethidium bromide solution (Genesee Scientific, San Diego, USA).

### MTT assay

The inhibition of cell proliferation was measured with the 3-(4,5-dimethylthiazol-2-yl)-2,5-diphenyltetrazolium bromide (MTT) assay kit (Cayman Chemical Company, Ann Arbor, USA). The principle of this method relies on the production of purple pigments by living cells upon cleavage of tetrazolium salt to formazan by their intracellular NAD(P)H-oxidoreductase, whereas such pigmentation is not produced by dead cells. Cells were seeded in a 96-well plate (Becton and Dickinson) at a density of 3 × 10 ^4^ cells/well in 100 μL of culture medium and incubated for 24 h at 37 °C with 5% CO _2_. Then, 10 μL of MTT reagent was added to each well. After mixing gently, the cells were incubated for 4 h at 37 °C with 5% CO _2_. After removal of the supernatant, 100 μL of crystal dissolving solution was added and mixed with the cell solution, and the sample was further incubated for 4 h at 37 °C with 5% CO _2_. Finally, the optical density at 550 nm was measured using a microplate reader (BIO-RAD, Benchmark, Hercules, USA).

The 50% inhibitory concentration (IC _50_) of Andro for each cell type was calculated using software ImageJ (ImageJ, RRID:SCR_003070).

### Cell cycle analysis

Cells (2×10 ^5^ cells) were collected by centrifugation (300×g at room temperature for 5 min), resuspended in 50 μL of PBS and fixed by 450 μL of 80% ethanol for more than 3 hours at -20°C. Cell pellets obtained by centrifugation (300×g, 5 min) were washed in 500 μL of PBS, incubated with 200 μL of Muse Cell Cycle Reagents (Merck Millipore Corporation, Darmstadt, Germany) in the dark for 30 min, and the cell cycle was measured by Muse Cell Analyzer (Merck Millipore Corporation) which uses miniaturized fluorescence detection and microcapillary cytometry to deliver single-cell analysis.

### Quantification of Annexin V-positive cell percentage

Apoptosis was detected using the Muse ^TM^ Annexin V and Dead Cell Assay Kit (Merck Millipore Corporation) in accordance with the manufacturer’s protocols. Briefly, cells were seeded in a 24-well plate dish (2×10 ^5^ cells/well) for 24 h and collected by centrifugation (300×g at 4°C for 5 min), resuspended in 100 μL of RPMI 1640 medium and then incubated with 100 μL fluorescently labeled Annexin V reagent at room temperature for 20 min. Percentages of all cells (alive plus dead) labeled with Annexin V (a label of apoptotic cells) and/or 7-AAD (7-Aminoactinomycin D; a fluorescent chemical compound with a strong affinity for DNA which is used as a label of late-apoptotic/dead cells) were measured using the Muse Cell Analyzer and expressed by dot plots.

### Caspase-3/7 activity analysis

Caspase-3/7 activity was analyzed using the Muse ^TM^ Caspase-3/7 Assay Kit (Merck Millipore Corporation) in accordance with the manufacturer’s protocols. Cells were seeded for 24 h at a concentration of 2×10 ^5^ cells/mL in a 24-well plate dish (Falcon). Cells were collected by centrifugation (300×g at 4°C for 5 min) and suspended in 50 μL of RPMI 1640 medium. Then, 5 μL of caspase-3/7 Reagent working solution (1 μL of Muse ^TM^ Caspase3/7 Reagent and 7 μL of 1× PBS) was added, and cells were incubated for 30 min at room temperature in the dark. Finally, 150 μL of 7-AAD working solution was added, and Caspase-3/7 activity and cell viability were measured using a Muse Cell Analyzer.

### Measurement of ROS production

ROS production was measured using the Muse ^TM^ Oxidative Stress Kit (Merck Millipore Corporation) according to the manufacturer’s protocols. Cells were collected by centrifugation (300×g at 4°C for 5 min), and then the supernatant was removed. Muse ^®^ Oxidative Stress Regent working solution (190 μL) was added into each tube containing 10 μL cell suspension. Cells were vortexed in the medium for 5 seconds and then incubated at 37°C for 30 min in the dark, and the percentage of ROS producing cells was determined by cytometry using the Muse Cell Analyzer.

### Measurement of mitochondrial membrane depolarization

The mitochondrial membrane depolarization was determined using the Muse ^TM^ MitoPotential Kit (Merck Millipore Corporation) according to the manufacturer’s protocols. Cells were collected by centrifugation (300×g at 20°C for 5 min) and then mixed with 100 μL of Assay Buffer, and 95 μL of MitoPotential working solution (Muse ^TM^ MitoPotential Dye diluted to 1:1000 in assay buffer). After incubating at 37°C for 20 min, 7-AAD reagent (5 μL) was added to each tube, and it was vortexed for 3 to 5 seconds. After incubation at room temperature for 5 min, percentages of all cells (alive plus dead cells) showing mitochondrial membrane depolarization and/or labeling with 7-AAD were measured using the Muse Cell Analyzer. The 7-AAD staining results of this experiment are not shown in the present study but were consistent with the 7-AAD staining results shown in Figure 4 and will be provided by the authors upon request.

### Statistical analysis

Data were analyzed using Excel software ( Microsoft Excel 365) and the Student’s *t*-test was used to assess statistical significance between the various treatments. Results were expressed as mean ± SD of three independent experiments. P < 0.05 was considered statistically significant.

## Results

### Effects of Andro on the cell viability

**Figure 1.**
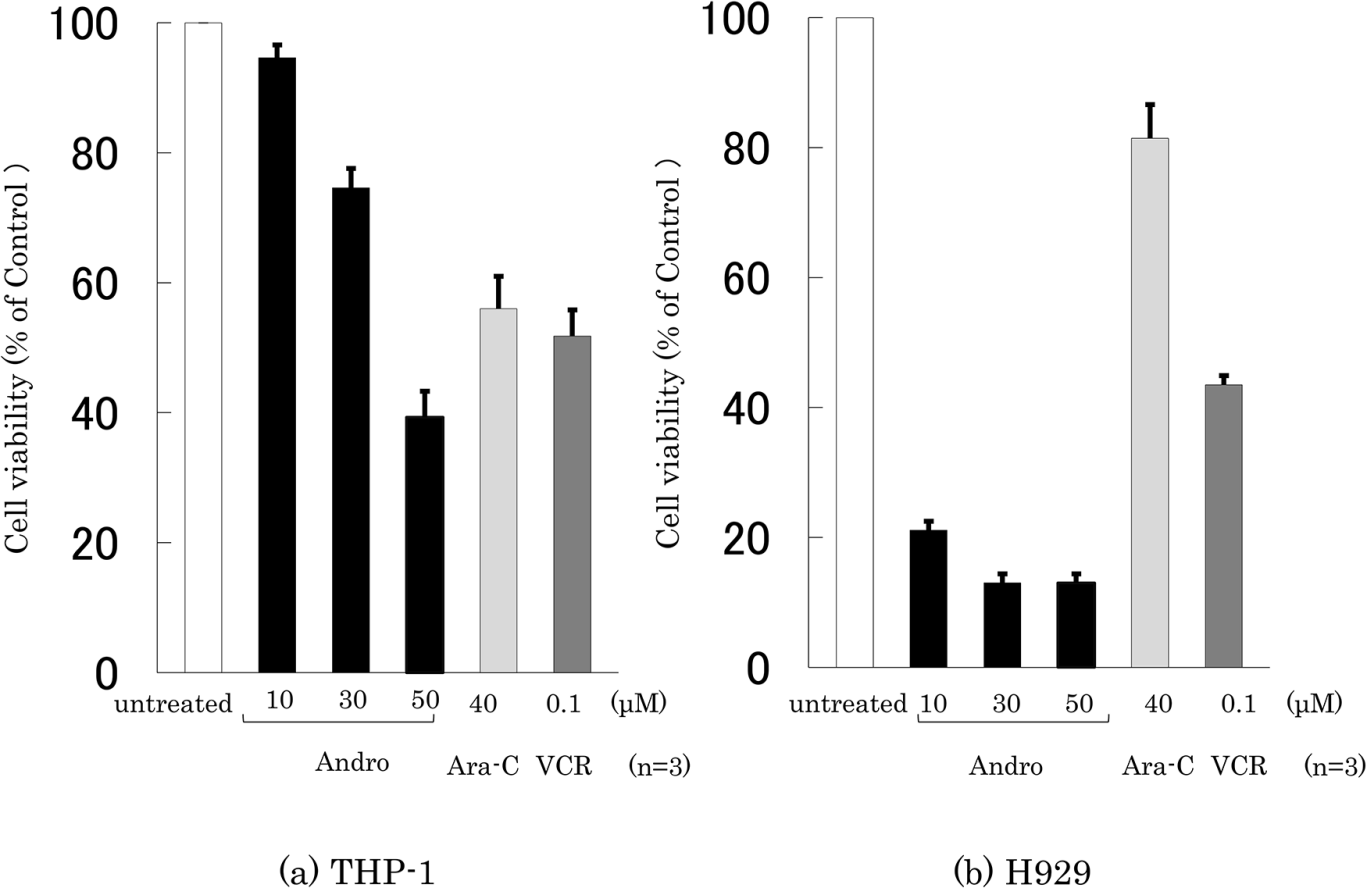
Assessment of cell viability after treatment for 24 h with Andro, Ara-C, or VCR. The y-axis values of the cell viability histograms represent the optical density (550 nm) in comparison with the control (set as 100%) as measured by MTT assay. The optical density markedly decreased after treatment with Andro (10, 30, 50 μM), Ara-C (40 μM), or VCR (0.1 μM) compared with untreated cells in THP-1 (a) and H929 (b) cells. The results are expressed as mean ± SD of three independent experiments.

The effects of Andro, Ara-C, and VCR on the viability of THP-1 and H929 cells were compared by incubating the cells for 24 h with or without an agent at the indicated concentrations, followed by an MTT assay ( Figure 1). Treatment with Andro (50 μM) reduced the viability of THP-1 and H929 cells to 39.2% and 13.0%, respectively, compared with untreated cells. The viability-reducing effect by Andro was concentration-dependent ( Figure 1) and its IC _50_ values for treating THP-1 and H929 cells were calculated as 31 μM and 8 μM, respectively.

Based on the therapeutic plasma concentrations of Ara-C and VCR for hematopoietic tumors ( Capizzi *et al.*, 1983; Nelson, 1982), Ara-C and VCR were used at 40 μM and 0.1 μM, respectively. They reduced the viability of THP-1 cells to 50-55%, whereas Ara-C only had a slight impact on H929 cells ( Figure 1). The viability-reducing effect of Andro on THP-1 cells was similar to that of Ara-C and VCR ( Figure 1a), whereas—at the concentrations used—Andro was markedly superior to Ara-C and VCR in reducing the viability of H929 cells ( Figure 1b).

### Effects of Andro on the morphology and DNA of the cells

**Figure 2.**
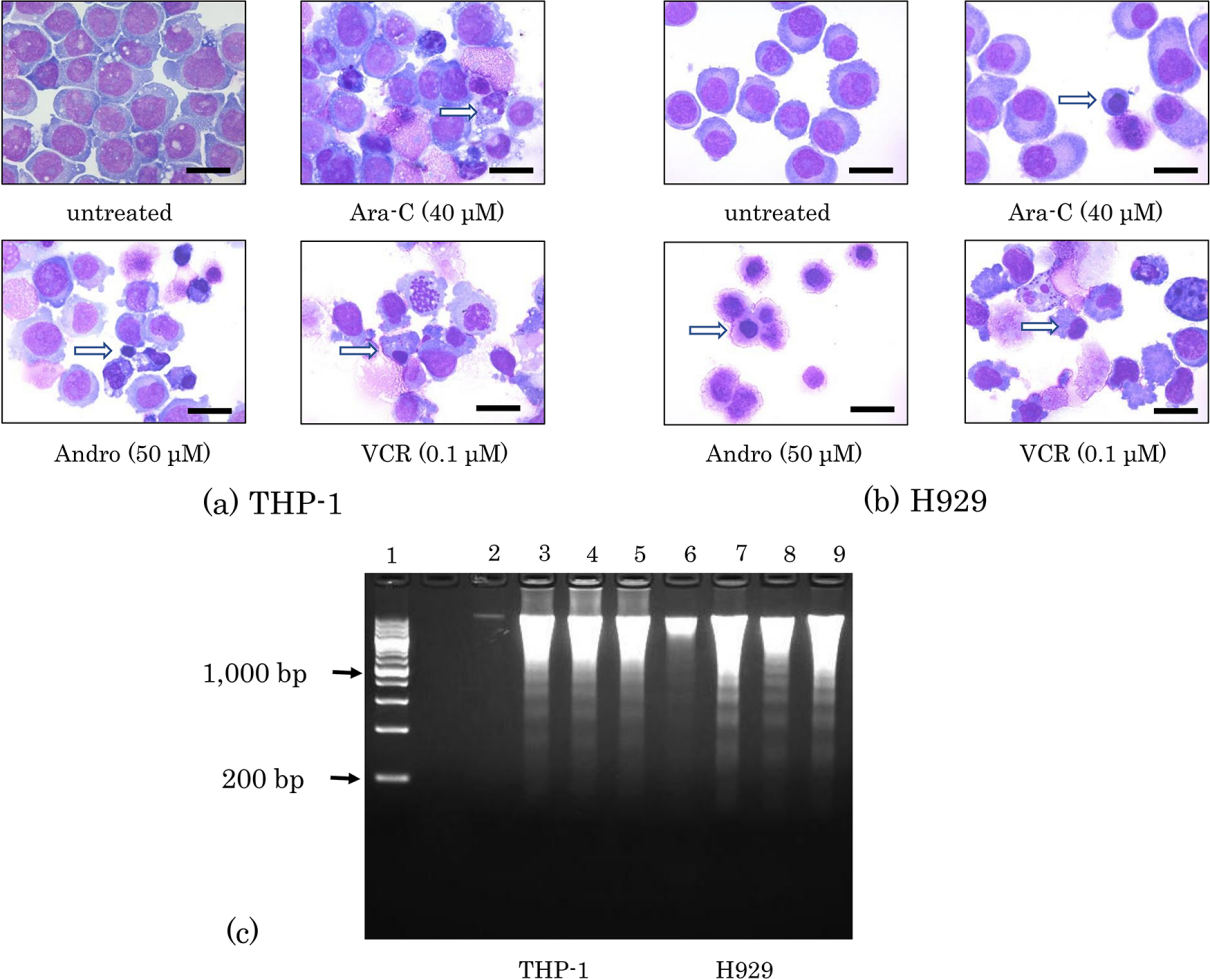
Morphological changes and DNA fragmentation induced by Andro, Ara-C, and VCR. Morphologies of THP-1 (a) and H929 (b) cells after 24 h of treatment with Andro, Ara-C, or VCR were compared with untreated cells after Wright-Giemsa staining. White arrows indicate cells showing nuclear condensation and black scale bars represent 20 μm. (c) Nuclear DNA fragmentation was revealed by agarose gel electrophoresis of DNA isolated after 24 h of treatment with Andro (50μM), Ara-C (40μM), or VCR (0.1μM) in THP-1 (lanes 2-5) and H929 cells (lanes 6-9). Lane 1, DNA size marker; lanes 2 and 6, untreated cells; lanes 3 and 7, cells treated with Andro; lanes 4 and 8, cells treated with Ara-C; lanes 5 and 9, cells treated with VCR.

Cellular shrinkage and nuclear condensation were observed in both THP-1 and H929 cells after treatment for 24 h with either Andro, Ara-C, or VCR ( Figure 2a,b). Andro induced both phenomena in almost all H929 cells ( Figure 2b). Furthermore, DNA isolation followed by agarose gel electrophoresis revealed that these treatments with Andro, Ara-C, and VCR each had induced nuclear DNA fragmentation in both THP-1 and H929 cells ( Figure 2c).

### Cell cycle analysis

**Figure 3.**
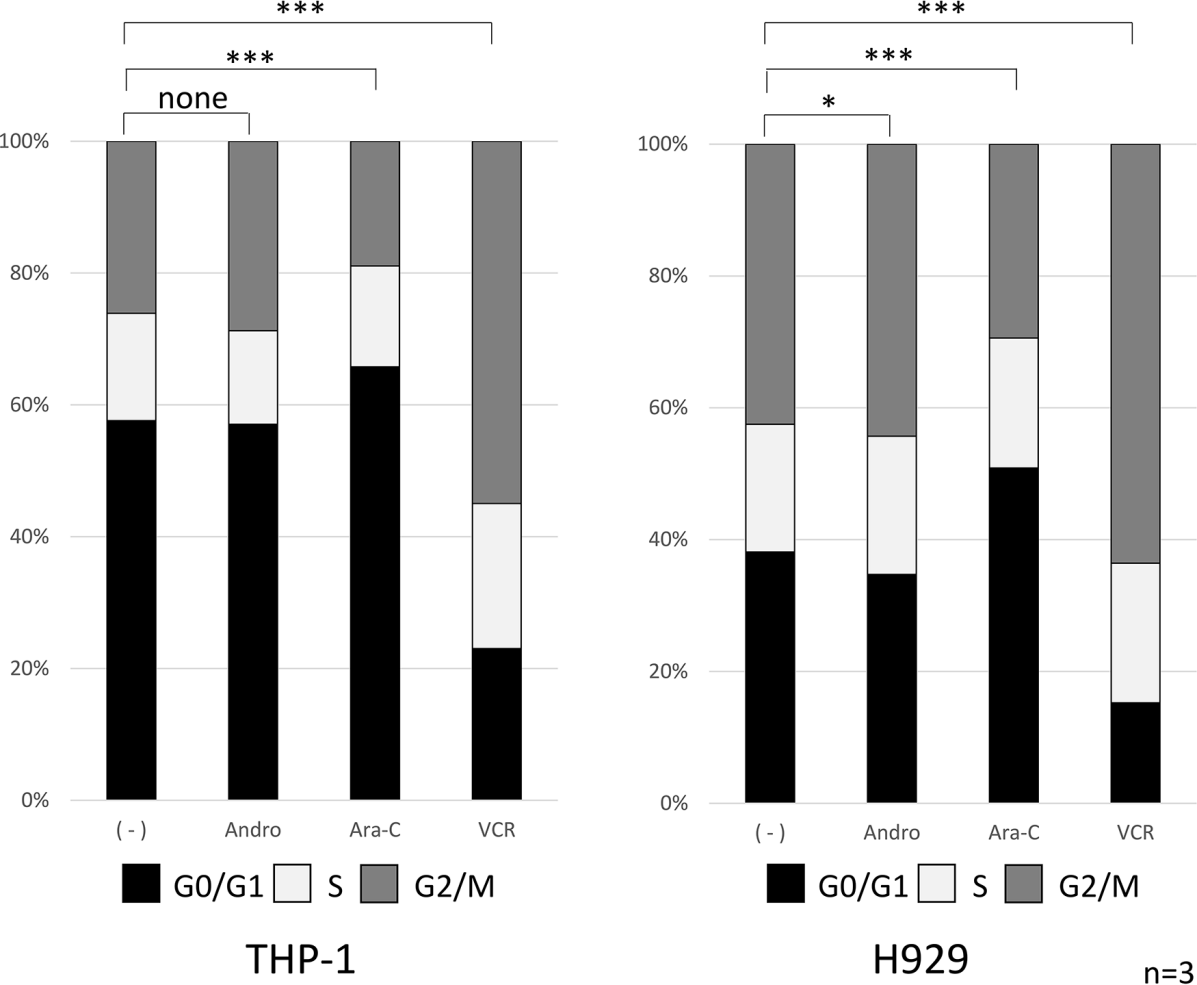
Cell cycle phase distribution of the cells treated with Andro, Ara-C, or VCR. Cell cycle phases of individual cells were measured after treatment for 24 h with Andro (50μM), Ara-C (40μM), or VCR (0.1μM) using the Muse Cell Analyzer. In contrast to Ara-C and VCR, treatment with Andro hardly affected the percentages of THP-1 or H929 cells found in the G0/G1, S, and G2/M phases. Percentages are expressed as mean of three independent experiments. For statistical analysis the percentages of cells in the G0/G1 phase were compared (*P < 0.05, **P < 0.01, ***P < 0.001).

The effects of 24 h treatment with Andro, Ara-C, or VCR on cell cycle progression were compared ( Figure 3). In the case of Andro, the percentages of cells in the G0/G1, S, and G2/M phases were very similar to those in untreated THP-1 and H929 cells. On the other hand, Ara-C treatment significantly increased the percentage of cells in the G0/G1 phase, in agreement with its known inhibition of DNA synthesis ( Li *et al.*, 2017). Likewise as expected, VCR significantly increased the percentage of cells in the G2/M phase, in agreement with its known inhibition of mitotic spindle formation ( Kothari *et al.*, 2016).

### Effects of Andro on the annexin V-positive rate of the cells

**Figure 4.**
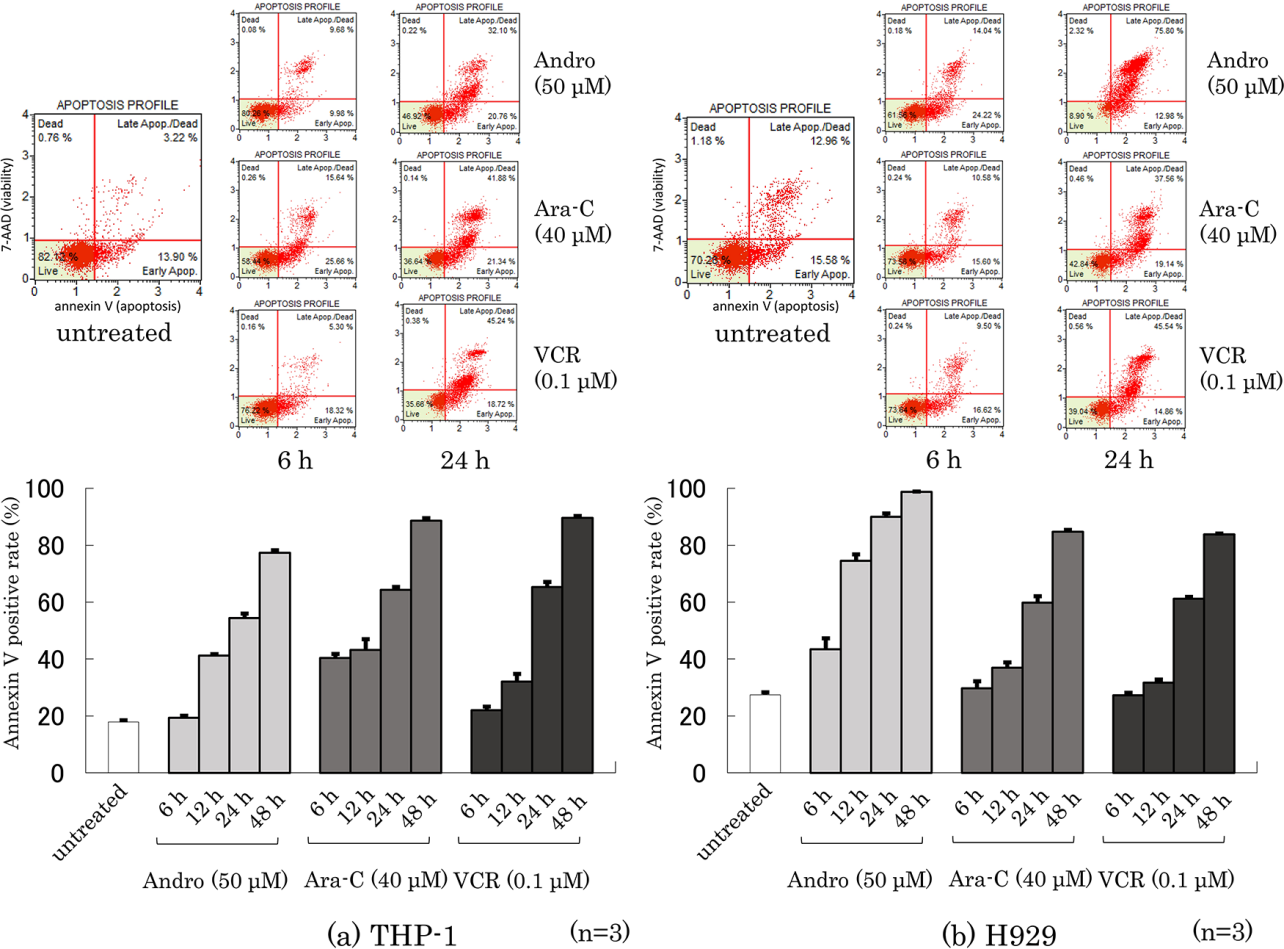
Rates of Annexin V-positive cells after treatment for 6~48 h with Andro, Ara-C, or VCR of THP-1 (a) and H929 (b) cells. Labeling with Annexin V and 7-AAD were analyzed by the Muse Cell Analyzer. The upper figures show representative dot plots in which the x-axis indicates Annexin V labeling and the y-axis indicates 7-AAD labeling. In the lower figures the Annexin V staining results are expressed as mean ± SD of three independent experiments.

Phosphatidylserine externalization from the inner to the outer cell membrane is a characteristic feature of apoptotic cell death which can be measured by annexin V-binding ( Demchenko, 2013). Dual labeling with annexin V and 7-AAD (a label for cells with permeabilized membranes such as late-apoptotic cells and dead cells) of THP-1 and H929 cells was performed after they had been treated for 6~48 h with Andro, Ara-C, or VCR. The percentages of annexin V-positive cells among THP-1 and H929 cells increased depending on their time of treatment with either anti-tumor agent ( Figure 4). Overall, higher percentages of annexin V-positive THP-1 cells were not found after treatment with Andro than with Ara-C or VCR ( Figure 4a), whereas Andro was markedly superior to Ara-C and VCR in inducing apoptosis in H929 cells ( Figure 4b). The 7-AAD-staining results, shown in the cell cytometry dot plots in the upper part of Figure 4, suggest that after 24 h treatment with Andro the majority of H929 cells were already dead, emphasizing the high toxicity of Andro for this cell type.

### Effects of Andro on the Caspase-3/7 activity of the cells

**Figure 5.**
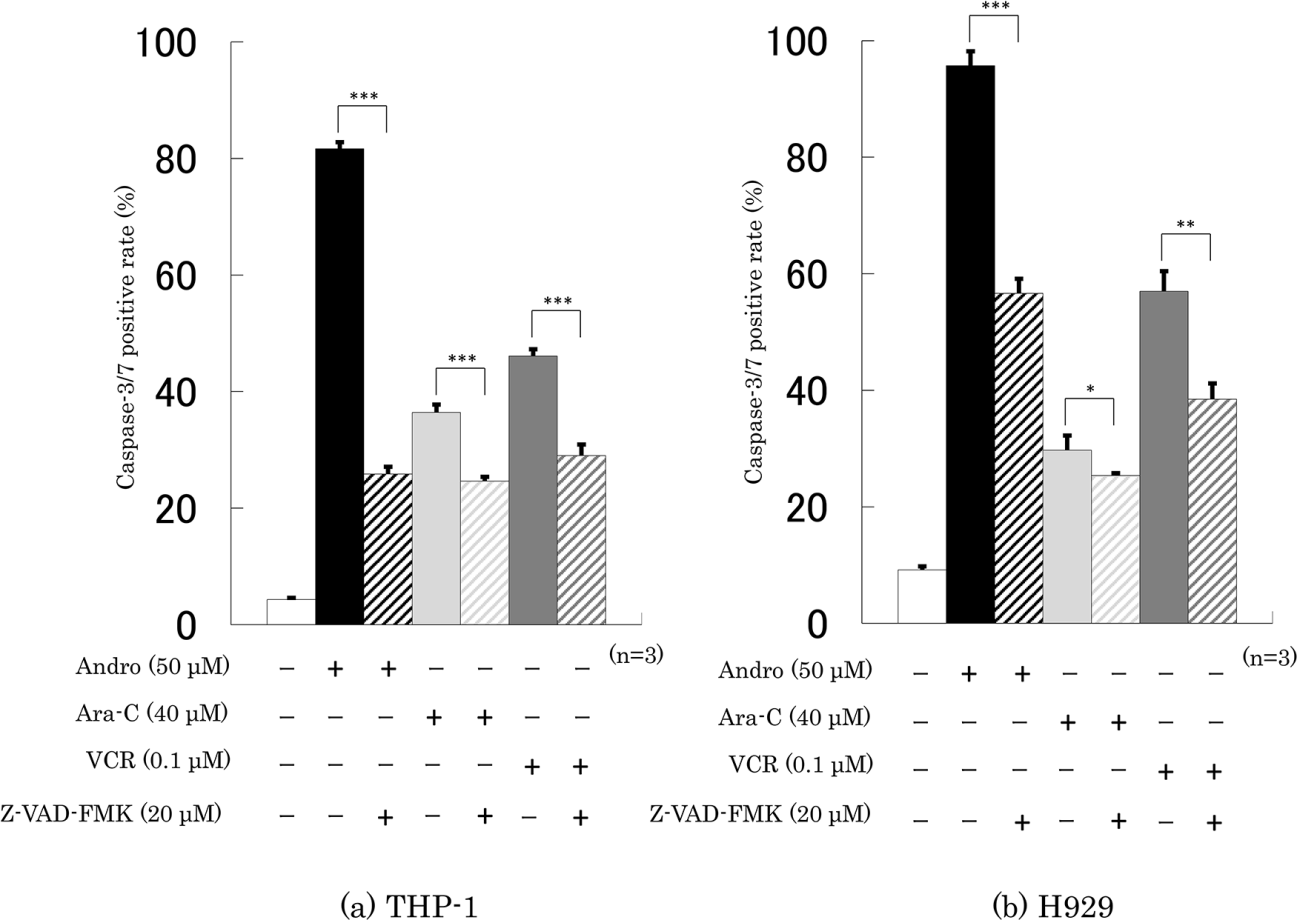
Treatment for 24 h with Andro, Ara-C, or VCR enhanced the caspase-3/7 activities in THP-1 (a) and H929 (b) cells, and the level of enhancement was reduced in the presence of the pan-caspase inhibitor Z-VAD-FMK. The results are expressed as mean ± SD of three independent experiments (*P < 0.05, **P < 0.01, ***P < 0.001, comparing with and without Z-VAD-FMK).

Treatment with Andro for 24 h increased the percentages of cells with caspase-3/7 activity from 4.3% to 81.7% in THP-1 cells ( Figure 5a) and from 9.2% to 95.7% in H929 cells ( Figure 5b). These increases were substantially higher than those induced with Ara-C or VCR treatments ( Figure 5). In the presence of a caspase inhibitor, Z-VAD-FMK, the Andro-induced caspase-3/7 positive rates of THP-1 and H929 cells were significantly lower, namely only 25.9% and 56.7%, respectively ( Figure 5). Z-VAD-FMK also significantly reduced, although not by as much, the enhancing effects of Ara-C and VCR on caspase 3/7 positive rates ( Figure 5).

### Effects of Andro on ROS production and mitochondrial membrane depolarization of the cells

**Figure 6.**
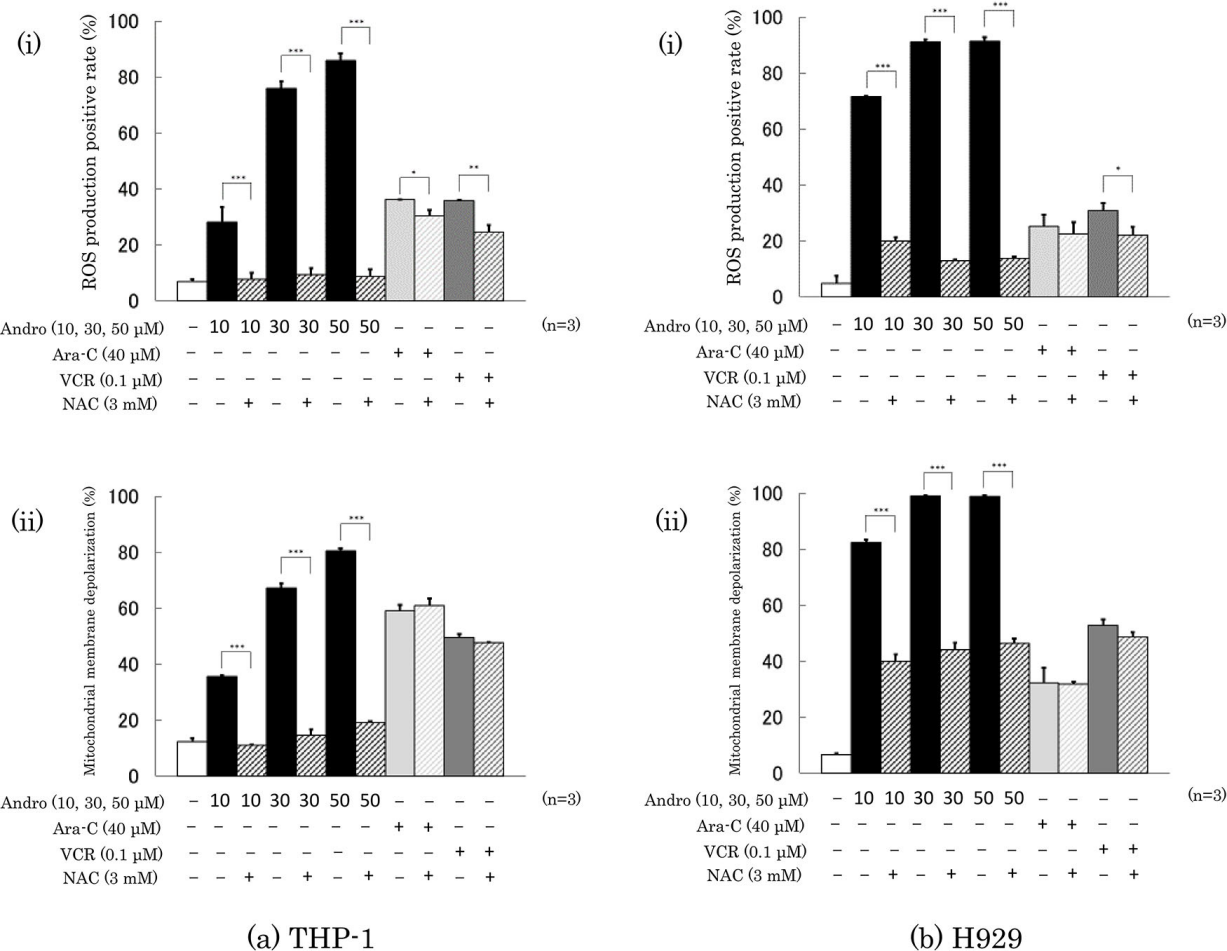
Effects of treatment for 24 h with Andro, Ara-C, or VCR on ROS production (a-i, b-i) and mitochondrial membrane depolarization (a-ii, b-ii) in THP-1 (a) and H929 (b) cells. The presence of the ROS production inhibitor NAC largely reduced the enhancing effects of Andro on both parameters in either cell type, whereas NAC had little or no impact on the effects of Ara-C and VCR. The results are expressed as mean ± SD of three independent experiments (*P < 0.05, **P < 0.01, ***P < 0.001, comparing with and without NAC).

Treatment with Andro (50 μM) for 24 h increased the percentage of ROS producing cells from 6.8% to 85.8% in THP-1 cells ( Figure 6a-i) and from 4.8% to 91.1% in H929 cells ( Figure 6b-i). Andro increased the ROS positive rates in a concentration-dependent manner, and in H929 cells even at 10 μM (the lowest concentration tested) the enhancing effect of Andro on ROS production was much higher than that of Ara-C or VCR. The ROS enhancing effect of Andro was largely abolished by the presence of ROS inhibitor NAC, whereas NAC only slightly reduced the ROS enhancing effects of Ara-C and VCR ( Figure 6a-i, b-i).

Consistent with the findings for ROS production, treatment with Andro (50 μM) for 24 h increased the percentages of cells with depolarized mitochondrial membranes from 12.3% to 80.5 % in THP-1 cells ( Figure 6a-ii) and from 6.5% to 98.8 % in H929 cells ( Figure 6b-ii). These Andro effects were concentration-dependent and even at 10 μM the effect of Andro on H929 cells was stronger than that of Ara-C or VCR. The presence of NAC significantly reduced the enhancement of mitochondrial membrane depolarization caused by Andro but hardly or not the effects of Ara-C or VCR ( Figure 6a-ii, b-ii).

**Figure 7.**
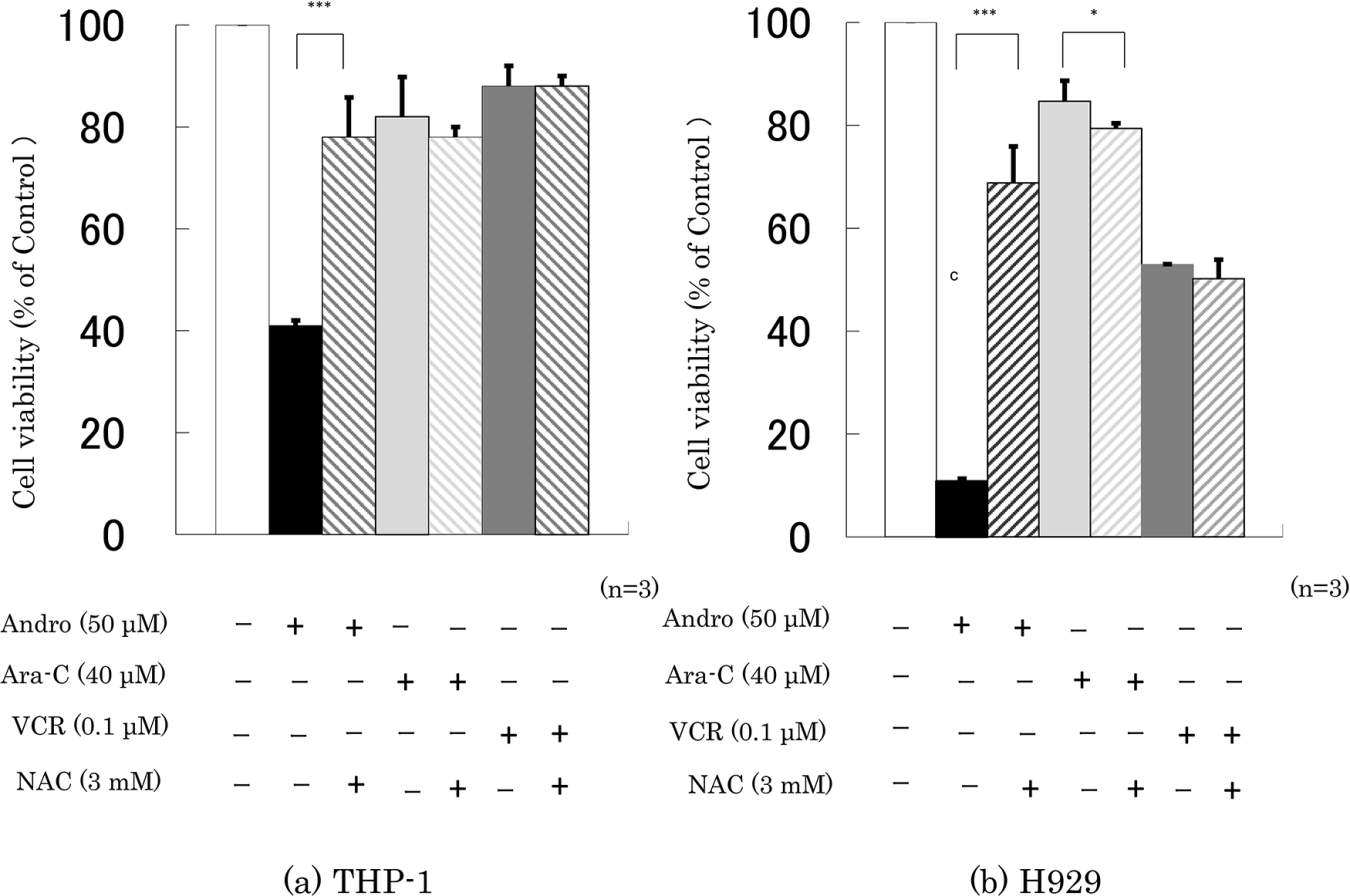
NAC largely reduces Andro’s effects on cell viability but has little impact on the effects of Ara-C and VCR. Cell viability was measured after 24 h treatment with Andro, Ara-C, or VCR of THP-1 (a) and H929 (b) cells in the presence or absence of the ROS production inhibitor NAC. The y-axis values represent the optical density (550 nm) in comparison with the control (set as 100%) as measured by MTT assay.

**Figure 8.**
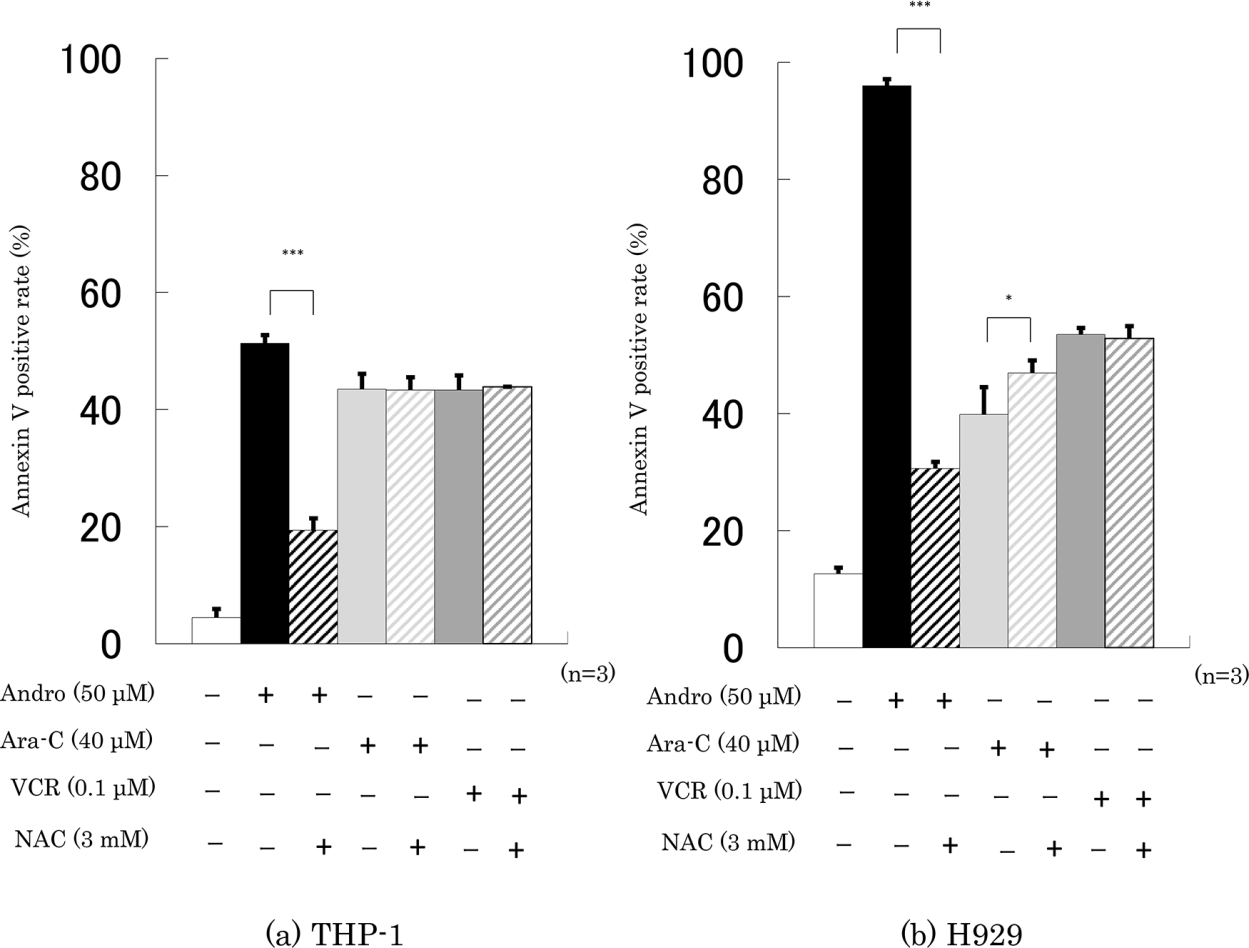
NAC largely reduces Andro’s stimulation of apoptosis but has little impact on the effects of Ara-C and VCR. Annexin V-positive rates were measured after 24 h treatment with Andro, Ara-C, or VCR of THP-1 (a) and H929 (b) cells in the presence or absence of the ROS production inhibitor NAC. The results are expressed as mean ± SD of three independent experiments (*P < 0.05, **P < 0.01, ***P < 0.001, comparing with and without NAC).

Finally, we checked whether the presence of NAC interfered with the effects of 24 h incubation with Andro, Ara-C, or VCR on cell viability and the percentage of annexin V-positive cells. It was found that NAC largely abolished the effects of Andro on both properties, especially in H929 cells, but had little or no impact on the effects of Ara-C or VCR ( Figures 7, 8).

## Discussion

The herb *Andrographis paniculate*, called “king of bitters” because of its extremely bitter taste, has been used for centuries for various medicinal purposes. The primary bioactive component of this medicinal plant is andrographolide, which is bitter and present in all parts of the plants but maximally (>2 % of dry weight) in the leaves ( Jarukamjorna and Nemoto, 2008; Sharma *et al.*, 2018). The present study confirms that andrographolide can be toxic for tumor cell lines, and for the first time determins its toxicity for the human multiple myeloma cell line H929. Another novel observation is that Andro exerts its toxic effect on the human leukemia monocytic cell line THP-1, and also on H929 cells, via induction of ROS-dependent apoptosis.

Apoptosis is a form of programmed cell death involving cascades of interactions ( Rossi and Gaidano, 2003; Schultz and Harrington, 2003). Andro-treated THP-1 and H929 cells showed typical symptoms of apoptosis, such as cellular shrinkage, nuclear condensation, DNA fragmentation, stainability with Annexin V, caspase 3/7 activation, and mitochondrial membrane depolarization. Notably, in the presence of NAC, an inhibitor of ROS production, the cytotoxic and apoptotic effects of Andro on THP-1 and H929 cells were largely abolished. The induction of ROS-dependent apoptosis by Andro has also been observed in other cancer cells such as a breast cancer cell line ( Banerjee *et al.*, 2016), a colon cancer cell line ( Khan *et al.*, 2018), and lymphoma cell lines and primary lymphoma ( Yang *et al.*, 2010). The levels of ROS production in THP-1 and H929 cells induced by Andro were much higher than induced by Ara-C and VCR, and—in sharp contrast to Andro—the cytotoxic/apoptotic effects of Ara-C and VCR were hardly sensitive to NAC. This implies a different mode of action and suggests that an additive anticancer therapeutic value might be achieved if Andro would be used in combination with agents such as Ara-C and/or VCR. While Ara-C is known to be a DNA polymerase inhibitor that inhibits DNA synthesis ( Li *et al.*, 2017), VCR inhibits mitosis by inhibiting microtubule polymerization ( Kothari *et al.*, 2016). Unfortunately, the mechanism by which Andro induces ROS-dependent apoptosis is still not understood (see below).

The (24 h) IC _50_ concentrations of Andro for reducing the cell viability of THP-1 and H929 cells were determined as 31 μM and 8 μM, respectively. These concentrations are far below the Andro concentrations at which normal cells are noticeably affected ( Banerjee *et al.*, 2016; Khan *et al.*, 2018) and somewhat lower than the (24 h) IC _50_ concentrations determined as 52 μM for colon cancer MDA-MB-231 cells ( Banerjee *et al.*, 2016), 40 μM for acute myeloid leukemic HL-60 cells ( Cheung *et al.*, 2005), and 60 μM for colon cancer HT-29 cells ( Khan *et al.*, 2018). For THP-1 cells, previously, low concentrations of Andro, namely ≤3 μM, were found to affect functional properties ( Gupta *et al.*, 2020; Ji *et al.*, 2005), but our current findings agree well with a report that the (72 h) LD _50_ concentration was ~20 μM ( Habtemariam, 2003). The high sensitivity to Andro that we observed for H929 viability is reminiscent of observations for other types of lymphoma cell lines, considering the (48 h) IC _50_ values reported for Ramos (Burkitt lymphoma) (20 μM), Granta (mantle cell lymphoma) (40 μM), HF-1 (follicular lymphoma) (15 μM), and SUDHL4 (diffuse large B-cell lymphoma) (30 μM) ( Yang *et al.*, 2010). In primary follicular lymphoma cells strong apoptotic effects were induced after 24 h incubation with only 5 μM Andro ( Yang *et al.*, 2010). High sensitivities of multiple myeloma cell lines have also been reported, as for the cell lines RPMI-8226 and U266 the Andro (48 h) LC _50_ concentrations were determined as10 μM and 8 μM, respectively ( Gunn *et al.*, 2011). Furthermore, a 72 h incubation with only 1 μM Andro reduced the viability of the multiple myeloma cell line OPM1 (RRID:CVCL_5210) to less than 70% ( Gao and Wang, 2016). In short, the Andro sensitivities that we observed for monocytic leukemia THP-1 and multiple myeloma H929 cells are in agreement with previous observations and emphasize that, in particular, multiple myeloma cells are very sensitive to Andro. From the viewpoint of potential therapeutic usage, this sensitivity is even more interesting given our finding that H929 cells are not very sensitive to Ara-C and VCR. Namely, this raises the hope that some tumor cells that are refractory to treatment with the common drugs Ara-C and VCR may be treated with Andro.

Andrographolide is considered nontoxic even at high doses ( Calabrese *et al.*, 2000; Sattayasai *et al.*, 2010), but its low aqueous solubility limits the plasma concentrations that can be readily achieved ( Pandey and Rao, 2018). However, steady-state blood concentrations of ~1.9 μM have been reported in humans taking ~1 mg andrographolide per kg body weight per day ( Panossian *et al.*, 2000), and this is expected to be within the therapeutically effective concentration range for Andro against multiple myeloma cells (see above).

Several studies found an effect of Andro on cell cycle phase distribution, and authors assumed that Andro induced cell cycle arrest ( Banerjee *et al.*, 2016; Cheung *et al.*, 2005; Dai *et al.*, 2017; Khan *et al.*, 2018). For example, for the gastric cancer cell line SGC7901 (RRID:CVCL_0520), it was reported that higher concentrations of Andro caused cell cycle arrest in the G2/M phase ( Dai *et al.*, 2017). In contrast, for the colon cancer cell line HT-29 (RRID:CVCL_0320), it was reported that low concentration of Andro caused significant cell cycle arrest in the G2/M phase, while higher Andro concentrations caused arrest in the G0/G1 phase ( Khan *et al.*, 2018). Meanwhile, for the acute myeloid leukemic cell line HL-60, Andro reportedly arrested the cells in G0/G1 phase ( Cheung *et al.*, 2005). In contrast to those studies, the present study did not find a notable effect of Andro on the cell cycle phase distribution of THP-1 and H929 cells. Our findings combined with the inconsistent cell cycle distribution effect of Andro reported for other tumor cell lines ( Banerjee *et al.*, 2016; Cheung *et al.*, 2005; Dai *et al.*, 2017; Khan *et al.*, 2018), and the fact that in those studies a true arrest probably cannot be considered as proven, suggest that Andro does not specifically target a specific step in cell cycle progression.

An important question is why cancer cells, compared to normal cells, can be more sensitive to the induction of ROS-mediated apoptosis. It may be related to mitochondria being the major source of reactive oxygen species (ROS) ( Orrenius, 2007; Vyas *et al.*, 2016) and the unusual properties of typical cancer cell mitochondria. Most cancer cells show an increased reliance on aerobic glycolysis (Warburg effect) ( Warburg, 1956) and many cancers, including multiple myeloma, show an enhanced biogenesis of mitochondria compared to normal cells ( Zhan *et al.*, 2017). Many cancers, including multiple myeloma (MM), retain more cytosolic iron to promote tumor cell growth, and higher cytosolic iron promotes oxidative damage due to its interaction with reactive oxygen species generated by mitochondria ( Zhan *et al.*, 2017).

The possibility to specifically target multiple myeloma cells for the induction of ROS-dependent apoptosis has already been shown for a number of agents. For example, *ex vivo* analysis showed that pharmacological-dosed ascorbic acid (PAA; ultra-high doses of vitamin C) selectively induced apoptosis in primary multiple myeloma cells while not significantly harming other bone marrow cells, and PAA-induced apoptosis in the multiple myeloma cell line OCI-MY5 could be inhibited by NAC ( Xia *et al.*, 2017). Furthermore, treatment with a mitochondrial-targeting agent decyl-triphenylphosphonium (10-TPP) increased intracellular steady-state pro-oxidant levels and apoptosis in multiple myeloma cell lines ( Schibler *et al.*, 2016); 10-TPP is a lipophilic agent that associates directly with mitochondria, likely with the inner membrane ( Murphy, 2008; Ross *et al.*, 2008; Schibler *et al.*, 2016). Dexamethasone, a glucocorticoid, is another hydrophobic lipophilic molecule that induced apoptotic cell death in multiple myeloma cell lines, and this effect could also be reduced by NAC ( Bera *et al.*, 2010); in sharp contrast, in normal cells dexamethasone was found to inhibit ROS generation ( Dandona *et al.*, 1999). As with Andro, the mechanism for the induction of apoptosis in tumor cells is likely not fully understood for any of the above three agents.

We speculate that the main effect of Andro involves a—yet to be identified—direct interaction with mitochondrial membranes, and that the end-effect of this interaction on the cell depends on the condition of the mitochondria and the redox status of the cell. Such a model would make it easier to explain why we and others find that Andro can induce apoptotic cell death (see above), whereas in other cell systems Andro has been proven to protect against oxidative stress and apoptosis (reviewed by Kishore *et al.*, 2017; Mussard *et al.*, 2019). A direct interaction of Andro—which is a lipophilic molecule ( Pandey and Rao, 2018)—with the mitochondrial membranes might also explain a protective effect of Andro against mitochondrial fission ( Geng *et al.*, 2019). Selective disruptive/apoptotic effects against only some mitochondria, such as in THP-1 and H929 cells, may also help explain why *Andrographis paniculate* can have Andro stored in different tissues without the plant itself being harmed. In plants, Andro appears to have defensive roles against bacteria ( Zhang *et al.*, 2020) and herbivores (Edwin *et al.*, 2016), although the mechanisms are not yet well understood. Possibly, the same features of Andro that evolved in plants to distinguish between self and enemy cells may also determine its different effects on cancerous and non-cancerous cells. The enormous medicinal potential of Andro means that future research to better clarify its functions and mechanisms is imperative.

## Conclusion

Andro induces ROS-dependent apoptosis in monocytic leukemia THP-1 and multiple myeloma H929 cells. This cytotoxic effect is mechanistically different from that of Ara-C and VCR, suggesting that these agents could have supplementary effects if used in combination therapies. H929 cells, in particular, are very sensitive to Andro while they are not very sensitive to Ara-C and VCR, underscoring Andro’s promise as a potential drug against multiple myeloma. Future studies must unravel the mechanisms of Andro’s anti-tumor effect in more detail. Our study supports that Andro may be a valuable addition to the growing palette of drugs that are available for chemotherapy against hematopoietic tumors.

## Data availability

### Underlying data

Harvard Dataverse: Doi et al. Table with individual data. https://doi.org/10.7910/DVN/W7UJMD ( Doi, 2021).

This project contains the following underlying data.

• Doi et al. data (this file lists the individual data that underlie the figures).

Data are available under the terms of the Creative Commons Zero “No rights reserved” data waiver (CC0 1.0 Public domain dedication).
