# Supplementary material for: Andrographolide, isolated from Andrographis paniculata , induces apoptosis in monocytic leukemia and multiple myeloma cells via augmentation of reactive oxygen species production
Source: F1000Res. 2022 Apr 29;10:542. Originally published 2021 Jul 6. [Version 3] doi: 10.12688/f1000research.53595.3 (PMC9069414; doi:10.12688/f1000research.53595.3)
Supplement: Peer review history

# Peer review: Reviewer response for version 2

**DOI:** 10.5256/f1000research.80787.r127602

**Published:** 2022-03-31

**Authors:** Leonardo Pereira Franchi

**Affiliations:** 1 Department of Biochemistry and Molecular Biology, Institute of Biological Sciences (ICB) 2, Samambaia Campus, Federal University of Goiás (UFG), Goiânia, GO, Brazil

The study entitled “Andrographolide, isolated from Andrographis paniculata, induces apoptosis in monocytic leukemia and multiple myeloma cells via augmentation of reactive oxygen species production” presented an *in vitr*o evaluation of a diterpenoid proposed as drug to treat cancer, especially for the myeloma cancer cell-lines THP-1 and H929. The manuscript is very informative and well-written. At this moment, only some minor points should be addressed before indexing:

**Abstract**

- Results - Please replace the word “dose” for “concentration”. 
- Conclusions - Please verify this sentence: *“The high toxicity for (thus forming: The high toxicity for H929 cells, by a mechanism that is different from that of Ara-C and VCR, is encouraging for further studies on the use of Andro against multiple myeloma.)”*.

**Methods**

- Materials - Please include IUPAC name of Andro. And also include a figure containing the chemical structure of Andro.
- MTT assay - How ImageJ was used to calculate IC50?

**Results**

*Figure 3:*

- Suggestion: Include the cell cycle histograms to easily visualization of the results obtained.
- Please include the error bars in the graphs.

*Figure 4:*

- Suggestion: Include the dotplot of cells 48h after the treatment.

**Discussion:**

- Page 11: *“These concentrations are far below the Andro concentrations at which normal cells are noticeably affected (Banerjee et al.,2016; Khan et al., 2018)”* - Please include the concentration range.

Is the work clearly and accurately presented and does it cite the current literature?

Yes

If applicable, is the statistical analysis and its interpretation appropriate?

Yes

Are all the source data underlying the results available to ensure full reproducibility?

Yes

Is the study design appropriate and is the work technically sound?

Yes

Are the conclusions drawn adequately supported by the results?

Yes

Are sufficient details of methods and analysis provided to allow replication by others?

Partly

Reviewer Expertise:

DNA damage, in vitro cytotoxicity, photodynamic therapy

I confirm that I have read this submission and believe that I have an appropriate level of expertise to confirm that it is of an acceptable scientific standard.

# Response

**Published:** 2022-04-06

**Authors:** Johannes M. Dijkstra

**Affiliations:** Fujita Health University, Japan

Dear Professor Franchi,

 Thank you for your time to review our study. We are very happy with your nice compliments. We have made the minor changes that you recommended.

 In detail (your comments are in Italic):

*Abstract*

*Results - Please replace the word “dose” for “concentration”.*

**Our response:** We have now changed this as requested.

*Conclusions - Please verify this sentence: “The high toxicity for (thus forming: The high toxicity for H929 cells, by a mechanism that is different from that of Ara-C and VCR, is encouraging for further studies on the use of Andro against multiple myeloma.)”.*

**Our response:** Thank you for pointing this out. This was a text error that occurred during editing. We have now corrected it.

*Methods*

*Materials - Please include IUPAC name of Andro. And also include a figure containing the chemical structure of Andro.*

**Our response:** In the Methods section, we have now included the IUPAC name of Andro. In the Discussion section, we have now added a new Figure 9 that shows the structure of Andro.

*MTT assay - How ImageJ was used to calculate IC50?*

**Our response: **We have now added the information that curve-fitting by the program “Rodbard” was used.

*Results*

*Figure 3:*

*Suggestion: Include the cell cycle histograms to easily visualization of the results obtained.*

*Please include the error bars in the graphs.*

**Our response:** We have now added error bars and representative cell cycle histograms in the modified Figure 3.

*Figure 4:*

*Suggestion: Include the dotplot of cells 48h after the treatment.*

**Our response:** We have now added the dotplots of the 48h results in the modified Fig. 4.

*Discussion:*

*Page 11: “These concentrations are far below the Andro concentrations at which normal cells are noticeably affected (Banerjee et al.,2016; Khan et al., 2018)” – Please include the concentration range.*

**Our response:** In the modified text, we now specify the Andro concentrations that were used in the different studies. It now says:

 “These concentrations are considerably below the Andro concentrations at which normal cells are noticeably affected, namely: Banerjee et al., 2016, found no notable effect on the growth of a normal breast epithelial cell line MCF-10A at 80 μM Andro; Khan et al., 2018, did not find significant growth effects on the normal intestine epithelial cell line IEC-6 even at 1024 μM Andro; and Yang et al., 2010, did not find apoptosis of normal lymphocytes at 50 μM Andro (those authors did not check higher concentrations). The 31 μM and 8 μM values are also lower than the (24 h) IC50 concentrations determined as 52 μM for colon cancer MDA-MB-231 cells (Banerjee *et al*., 2016), 40 μM for acute myeloid leukemic HL-60 cells (Cheung et al., 2005), and 60 μM for colon cancer HT-29 cells (Khan *et al.*, 2018).

 We thank you again for all your efforts and the appreciation of our work.

 Sincerely,

 Also on behalf of the other authors,

 Hidehiko Akiyama

# Peer review: Reviewer response for version 1

**DOI:** 10.5256/f1000research.57001.r90836

**Published:** 2021-08-19

**Authors:** Christian Bailly

**Affiliations:** 1 OncoWitan, Lille, France

This is another study of the effect of the diterpenoid lactone andrographolide on the growth of cancer cells and the induction of cell death. Two cell lines (THP-1 and H929) were used and the effects of Andro compared to those of the Vinca alkaloid vincristine and cytarabine (Ara-C). The data are correctly presented but there is nothing really new or exciting in this study. The ROS-dependent pro-apoptotic activity of Andro has been already reported in several studies with other cancer cell lines. Showing the same type of data/effects with additional cell lines is not a major discovery. There is a lack of innovation and originality, despite the quality of the data.

 Moreover, the conclusion is excessively optimistic and excessive. Andro is known for a long time and the development of this compound has failed, due to an unfavorable benefit/risk ratio (low bioavailability, toxicity, etc.). The limitations should be more clearly indicated.

 The literature coverage could be improved, to cite recent reviews about Andro and cancer.

Is the work clearly and accurately presented and does it cite the current literature?

Partly

If applicable, is the statistical analysis and its interpretation appropriate?

Yes

Are all the source data underlying the results available to ensure full reproducibility?

Yes

Is the study design appropriate and is the work technically sound?

Yes

Are the conclusions drawn adequately supported by the results?

Partly

Are sufficient details of methods and analysis provided to allow replication by others?

Yes

Reviewer Expertise:

Anticancer pharmacology

I confirm that I have read this submission and believe that I have an appropriate level of expertise to confirm that it is of an acceptable scientific standard.
